# Supplementary material for: Glycochenodeoxycholate Affects Iron Homeostasis via Up-Regulating Hepcidin Expression
Source: Nutrients. 2022 Aug 2;14(15):3176. doi: 10.3390/nu14153176 (PMC9370805; doi:10.3390/nu14153176)
Supplement: Supplementary file 1 [file nutrients-14-03176-s001.zip › nutrients-1819816-supplementary.pdf]

Table S1. Primers used for RT-qPCR analysis.

Samples	Gene	Forward primer	Reverse primer
**HepG2 cell**	**HAMP**	**CTGACCAGTGGCTCTGTTTTC**	**GAAGTGGGTGTCTCGCCTC**
	IL-6	TTCCACGAAGTGACAGTGTGA	GCACGGTAGAAAAGGAAGGGT
	BMP2	AATGCAAGCAGGTGGGAAAG	GCTGTGTTCATCTTGGTGCA
	BMP4	AGCAGCCAAACTATGGGCTA	TGGTTGAGTTGAGGTGGTCA
	BMP6	AAGGCTGGCTGGAATTTGACATCACG	GGTAGAGCGATTACGACTCTGTTGTC
	ALK2	GCCTGGAGCATTGGTAAGC	CTGCCCACAGTCCTTCAAG
	ALK3	GCTATGATCATCTTCTCCAGCTGC	TGGCAATAGTTCGCTGAACCAA
	ActRIIA	GCCAGCATCCATCTCTTGAAGAC	GATAACCTGGCTTCTGCGTCGT
	ActRIIB	CGCTTTGGCTGTGTCTGGAAGG	CAGGTTCTCGTGCTTCATGCCA
	HJV	GCTAACCCTGGGAACCATGTG	CCCAACACAGAGCTGCAGGT
	TGR5	CTGGCCCTGGCAAGCCTCAT	CTGCCATGTAGCGCTCCCCGT
	FXR	GCAGCCTGAAGAGTGGTACTCTC	CATTCAGCCAACATTCCCATCTC
	β-Actin	AACTGGTCTCAAGTCAGTGTACAGG	TCCCCCAACTTGAGATGTATGAAG
Mice	DMT1	GTGATCCTGACCCGGTCTATCG	TGAGGATGGGTATGAGAGCAAAGG
	TNF-α	CACAAGATGCTGGGACAGTGA	TCCTTGATGGTGGTGCATGA
	IL-6	CTGCAAGAGACTTCCATCCAGTT	AGGGAAGGCCGTGGTTGT
	TFR1	AACTTACCCATGACGTTGATTGAACC	ACAGCCACTGTAGACTTA-GACCCATATC
	β-Actin	GGCTGTATTCCCCTCCATCG	CCAGTTGGTAACAATGCCATGT
	HAMP	CTGCCTGTCTCCTGCTTCTC	AGATGCAGATGGGGAAGTTG

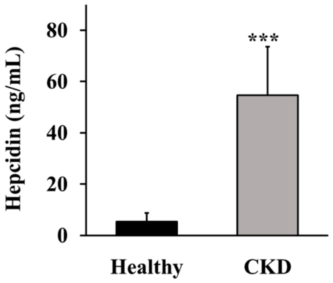

Figure S1. Concentration of serum hepcidin in 10 healthy subjects and 20 CKD patients. Values were shown as means ± SD. ***, $p < 0.001$, group of CKD patients vs. group of healthy subjects.

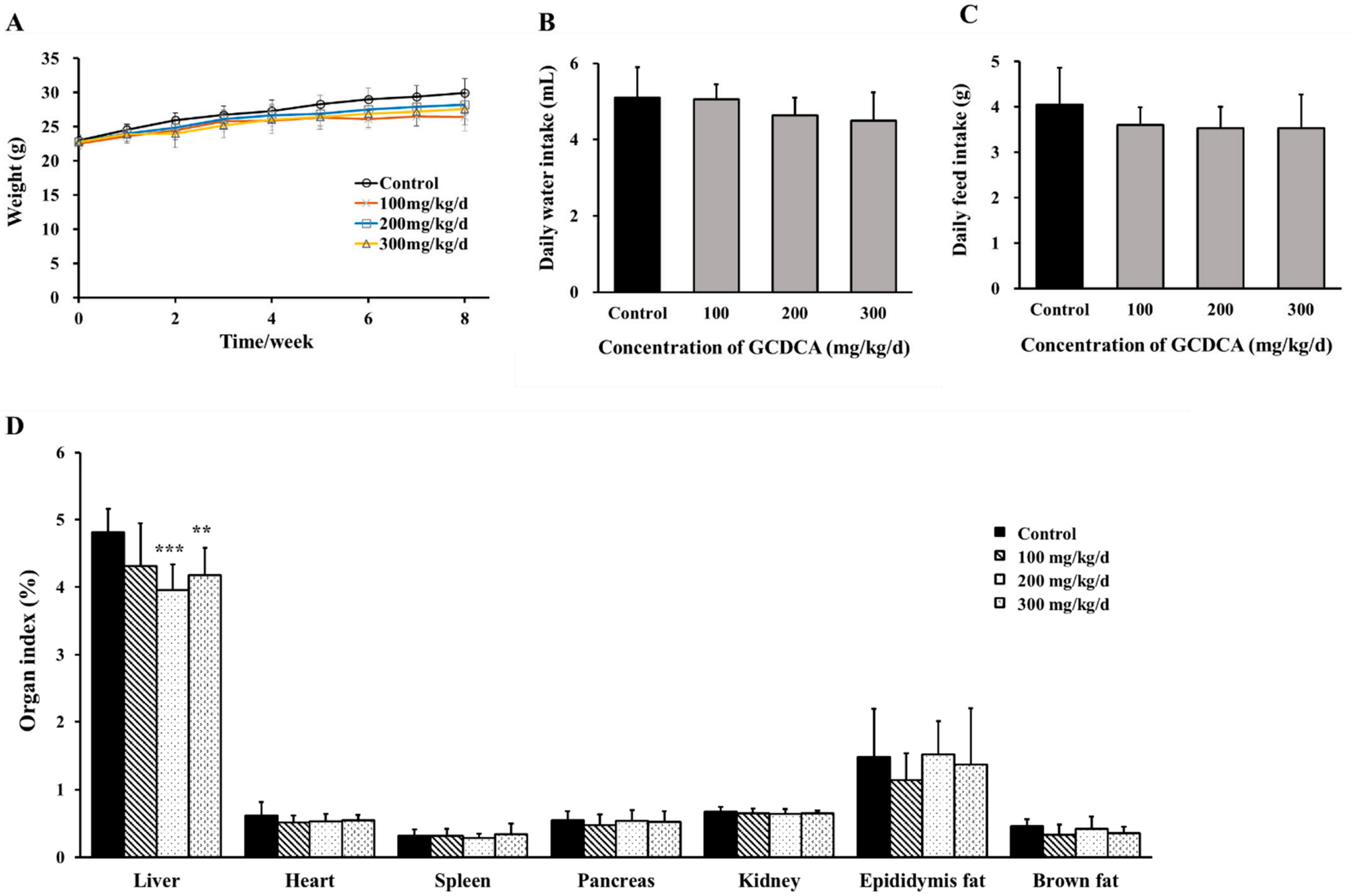

Figure S2. Effect of GCDCA administration on body weight and organ index in mice. (A) Effect of GCDCA administration on body weight of mice at treatment concentrations of 0, 100, 200 and 300 mg/kg/d. (B) Effect of GCDCA administration on daily water intake of last three days before the end of experiment. (C) Effect of GCDCA administration on daily feed intake of last three days before the end of experiment. (D) Effect of GCDCA administration on organ index of mice at concentrations of 0, 100 mg/kg/d, 200 mg/kg/d and 300 mg/kg/d. **, $p < 0.01$, GCDCA treatment group vs. control group. ***, $p < 0.001$.
